# Coenzyme Q_10_ Deficiency and Elevated LEAK Mitochondrial Respiration as Potential Heart Failure Markers in Ebstein Anomaly

**DOI:** 10.3390/ijms27083347

**Published:** 2026-04-08

**Authors:** Filip Klaučo, Iveta Šimková, Zuzana Sumbalová, Tereza Hlavatá, Monika Kaldarárová, Guillermo López-Lluch, Anna Gvozdjáková

**Affiliations:** 1 Department of Cardiology and Angiology, Adult Congenital Heart Disease Division, Slovak Medical University, National Institute of Cardiovascular Diseases, Pod Krásnou Hôrkou 1, 833 48 Bratislava, Slovakia; filip.klauco@gmail.com (F.K.); simkova.iveta@gmail.com (I.Š.);; 2 Institute of Medical Chemistry, Biochemistry and Clinical Biochemistry, Faculty of Medicine, Comenius University in Bratislava, Sasinkova 2, 811 08 Bratislava, Slovakia; 3 Department of Physiology, Anatomy and Cell Biology, Andalusian Centre of Developmental Biology (CABD-CSIC-JA), Universidad Pablo de Olavide, Carretera de Utrera Km. 1, 410 13 Seville, Spain; glopllu@upo.es

**Keywords:** Ebstein anomaly, platelets, mitochondrial bioenergetics, OXPHOS, FAO, reprogramming, coenzyme Q_10_, γ-tocopherol, personalized treatment

## Abstract

Congenital heart diseases (CHDs) are characterized by profound metabolic remodeling of mitochondrial pathways. However, data regarding mitochondrial respiration, oxidative phosphorylation (OXPHOS), and fatty acid oxidation (FAO) in patients with Ebstein anomaly (EA) are currently unavailable. This study evaluated 14 EA patients and 18 healthy volunteers. In accordance with the 2020 ESC guidelines, patients were stratified into two cohorts: EA-0 (patients currently without an indication for intervention) and EA-1 (patients meeting Class Ia or IIb indications for surgical intervention). Platelet OXPHOS and FAO parameters were determined simultaneously via high-resolution respirometry. CI-linked LEAK respiration (substrates: pyruvate and malate) and FAO-linked LEAK respiration (substrates: octanoylcarnitine and malate) were significantly elevated in EA patients. Furthemore, the EA-1 group showed significantly lower coenzyme Q_10_ (CoQ_10_) and γ-tocopherol levels than EA-0. Differences in the measured parameters between groups suggest a state of myocardial adaptation and transient metabolic reprogramming in EA-0 patients, whereas in EA-1 patients, a significant change in mitochondrial metabolism and bioenergetics was found. We hypothesize that increased platelet LEAK mitochondrial respiration and CoQ_10_ deficiency could be key signals of mitochondrial reprogramming and serve as potential biomarkers for right ventricular dysfunction. The analysis of platelet mitochondrial bioenergetics represents a novel area of translational mitochondrial cardiology, contributing to personalized diagnostics, risk stratification and optimal surgical timing in EA patients.

## 1. Introduction

Translational mitochondrial cardiology applies basic scientific research to clinical practice [1]. The heart muscle activity depends on continuous energy supply by mitochondria, which constitute 30–40% of the total cardiomyocyte volume [2]. In a healthy adult heart, mitochondria produce approximately 6 kg of adenosine triphosphate (ATP) daily [3]. ATP is produced mainly by fatty acid oxidation (FAO), accounting for 40–60%, by glucose oxidation (20–40%), by ketone body oxidation (10–15%), and by amino acid oxidation (1–2%). Beyond energy production, mitochondria regulate numerous metabolic processes and serve as the primary site for reactive oxygen species (ROS) production. Impaired mitochondrial bioenergetics, mitochondrial DNA (mtDNA) damage, and the dysregulation of the balance between ROS production and antioxidant capacity are considered key factors in the development of many diseases, including cardiovascular diseases [1,2,4,5,6,7].

In cardiovascular diseases, including congenital heart diseases (CHDs), chronic pressure and volume overload lead to significant reductions in mitochondrial FAO and ATP production [8,9,10,11,12]. Ebstein anomaly (EA) is a rare CHD, characterized by morphological and functional malformations of the tricuspid valve primarily involving the apical displacement of the septal and posterior leaflets [13]. Severe tricuspid regurgitation, secondary to this malformation, causes chronic volume overload, progressive right ventricular (RV) dilation and systolic dysfunction. This adverse remodeling is associated with an elevated risk of heart failure, arrhythmias, and sudden cardiac death [14,15]. Prognosis and the quality of life for these patients depend heavily on preserving normal RV function [16,17].

While the 2020 ESC Guidelines provide a standard framework for the management of EA, emerging evidence underscores the necessity for a more proactive approach and earlier intervention. Given that surgical repair is the only definitive therapeutic approach, identifying molecular biomarkers that signal early ventricular exhaustion is crucial. This would allow for optimized surgical timing before the onset of irreversible structural remodeling. Currently, personalized diagnostic and therapeutic approaches are being developed for the early detection of worsening symptoms in patients with CHD.

To study mitochondrial bioenergetics in EA patients, we used circulating platelets isolated from the bloodstream. In recent years, the determination of platelet mitochondrial function has become an established tool in translational medicine [18]. Analyzing the mitochondrial respiration and energy profiles of platelets and peripheral blood mononuclear cells (PBMCs) represents a novel research area with potential for developing bioenergetic markers to improve diagnosis, therapy, and prognosis of cardiovascular diseases [4,19,20]. Mitochondrial bioenergetics measurements using RP1 and RP2 protocols show great potential for the rapid detection of disease progression and personalized treatment monitoring [1,21].

Coenzyme Q_10_ (CoQ_10_) is an essential component of the mitochondrial respiratory chain, necessary for ATP production via oxidative phosphorylation. Its reduced form, ubiquinol, has strong anti-oxidative properties that protect cells from ROS-induced damage whereas the oxidized form is needed to accept electrons from mitochondrial complexes and other enzymes [22]. CoQ_10_ deficiency has been observed in various pathophysiological conditions, including heart failure [23,24,25]. Our pilot study confirmed reduced endogenous CoQ_10_ levels in the platelets of patients with EA [26,27]. Despite these findings, the specific bioenergetic mechanisms driving RV dysfunction in EA are poorly understood.

In this study, we tested the hypothesis that platelet mitochondrial respiration and energy production are affected in patients with Ebstein anomaly. We employed high-resolution respirometry (HRR) by RP1 and RP2 protocols and determined endogenous CoQ_10_ levels by the high-performance liquid chromatography (HPLC) method.

## 2. Results

Patients with EA and healthy volunteers were included in the study. In accordance with the 2020 ESC guidelines [28], patients were stratified into two cohorts: EA-0 (patients currently without an indication for intervention) and EA-1 (patients meeting Class Ia or IIb indications for surgical intervention).

### 2.1. Characteristics of Patients with Ebstein Anomaly

In this study, 14 adult patients with Ebstein anomaly (2 male and 12 female) and 18 healthy controls (10 male and 8 female) were included. The mean age of the EA patients was 49.2 ± 11.3 years; the mean BMI was 24.2 ± 6.69 kg/m^2^. The mean age of the control group was 50.4 ± 12.2 years; the mean BMI was 25.9 ± 3.45 kg/m^2^. NYHA classes of EA patients were as follows: NYHA I (*n* = 2), NYHA II (*n* = 9), NYHA III (*n* = 3). Comorbidities included arterial hypertension (*n* = 7), coronary artery disease (*n* = 1), arrhythmia (*n* = 8), dyslipidemia (*n* = 6), chronic obstructive pulmonary disease/bronchial asthma (*n* = 2), hepatopathy (*n* = 2), and chronic kidney disease (*n* = 1). Medications included ACE inhibitors (*n* = 8), beta-blockers (*n* = 7), aldosterone blockers (*n* = 4), anticoagulants and antiplatelets (*n* = 6).

### 2.2. Metabolic Parameters in Patients with Ebstein Anomaly

The monitored metabolic parameters were compared with reference values, and the data between the EA-0 and EA-1 patient groups were statistically evaluated. All measured values were within the reference ranges of the individual parameters. Statistically significant differences were found only in total bilirubin levels (*p* = 0.0037) and in hemoglobin concentrations (*p* = 0.02), where higher values were observed in the EA-1 vs. EA-0 group (Table 1).

### 2.3. Platelet Mitochondrial Respiration in Patients with Ebstein Anomaly by Protocol RP1

**Figure 1:**
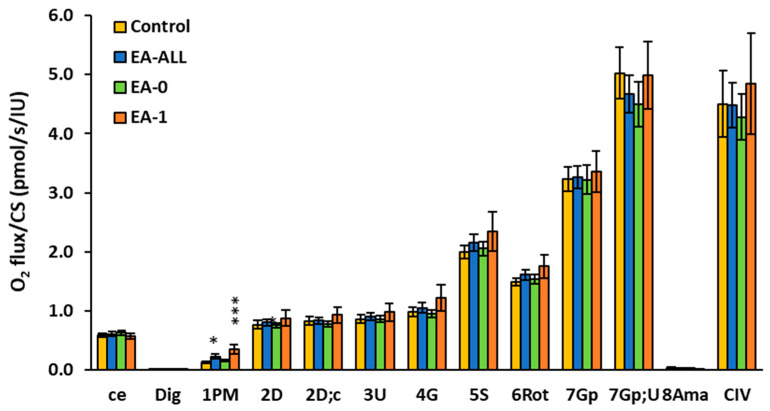
Platelet mitochondrial respiration and OXPHOS in patients with Ebstein anomaly by protocol RP1. Legend: statistical significance, * *p* < 0.05; *** *p* < 0.001. Subjects: Control group (C), group of all patients with Ebstein anomaly (EA-All), EA patients with non-failing heart (EA-0) and EA patients with failing heart (EA-1). The respiration was measured in freshly isolated platelets following substrate–uncoupler–inhibitor titration (SUIT) reference protocol RP1 [29] and evaluated as O_2_ flux per mitochondrial marker citrate synthase activity. The graph shows the mean ± SEM of the respiratory capacities after the titration step indicated on the x-axis: ce—oxygen consumption in intact platelet; Dig—digitonin—platelet permeabilization; 1PM—mitochondrial respiration after pyruvate plus malate addition; 2D—ADP addition—ATP production (CI-linked OXPHOS); 2D;c—cytochrome *c* addition for testing the integrity of outer mitochondrial membrane; 3U—uncoupler FCCP; 4G—glutamate; 5S—succinate; 6Rot—rotenone (an inhibitor of CI); 7Gp—glycerophosphate; 7Gp;U—uncoupler; 8Ama—Antimycin A (an inhibitor of CIII); CIV—cytochrome *c* oxidase. For CIV measurement, artificial substrates TMPD and ascorbate were added, and, after stabilization of respiration, CIV was inhibited by sodium azide. CIV activity was evaluated based on the difference in O_2_ flux with TMPD + ascorbate and O_2_ flux after the addition of sodium azide. See the Methods section for details.

The results of platelet mitochondrial respiration in patients with Ebstein anomaly by protocol RP1 are presented in Figure 1 and Table 2. Oxygen consumption in intact platelets *(ce*) was slightly changed in EA-All patients vs. the control group (to 104%). After permeabilization of platelets with digitonin, CI-linked mitochondrial respiration with substrates pyruvate and malate (1PM) in the EA-All group was statistically significantly increased (*p* < 0.05) to 170% compared to the control group. This parameter was increased in the EA–0 group to 120% and in the EA–1 group to 259% (*p* = 0.001). These results reflect pathological elevation in CI-linked LEAK mitochondrial respiration by substrate pyruvate with malate, which may participate in reprogramming of cell metabolism. In EA-All patients, the CI-linked OXPHOS capacity after ADP addition (respiration associated with ATP production) (2D), as well as after cytochrome *c* addition (2D;c), was not significantly changed in comparison with control data. After uncoupler addition (3U), CI-linked noncoupled mitochondrial respiration (CI-linked electron transfer (ET) capacity) was similar to the control group. After CI-linked substrate glutamate addition (4G), the ET capacity was slightly stimulated only in the EA-1 group (to 125%) vs. control values. After the addition of substrate succinate (5S), the respiration representing the ET capacity with CI&CII-linked substrates was slightly stimulated (to 118%) only in the EA-1 group vs. control data. After rotenone (6Rot) addition—an inhibitor of CI—values of respiration were similar to the control group. The mitochondrial respiration after glycerophosphate and additional uncoupler titration (7Gp;U) was decreased in EA-0 patients, reaching 89% of control values, which could indicate an impairment in the glycerophosphate pathway. In the EA-1 group, data were similar to the control group. The activity of CIV complex (cytochrome *c* oxidase) was slightly decreased in the EA-0 group (to 95%); in the EA-1 group, it increased slightly (to 108%) in comparison with control values (Figure 1, Table 2). These differences vs. control values were statistically non-significant.

### 2.4. The Efficiency of OXPHOS in EA Patients by Protocol RP1

For the efficiency of OXPHOS in platelet mitochondria, parameter (1-L/P) was used. The range of *P-L* OXPHOS efficiency is from 0 to 1; a value of 1 indicates the highest efficiency of OXPHOS. The *P-L* OXPHOS efficiency associated with Complex I of the mitochondrial respiratory chain was calculated using the measured values: (2D − 1PM)/2D = 1 − 1PM/2D [30].

**Figure 2:**
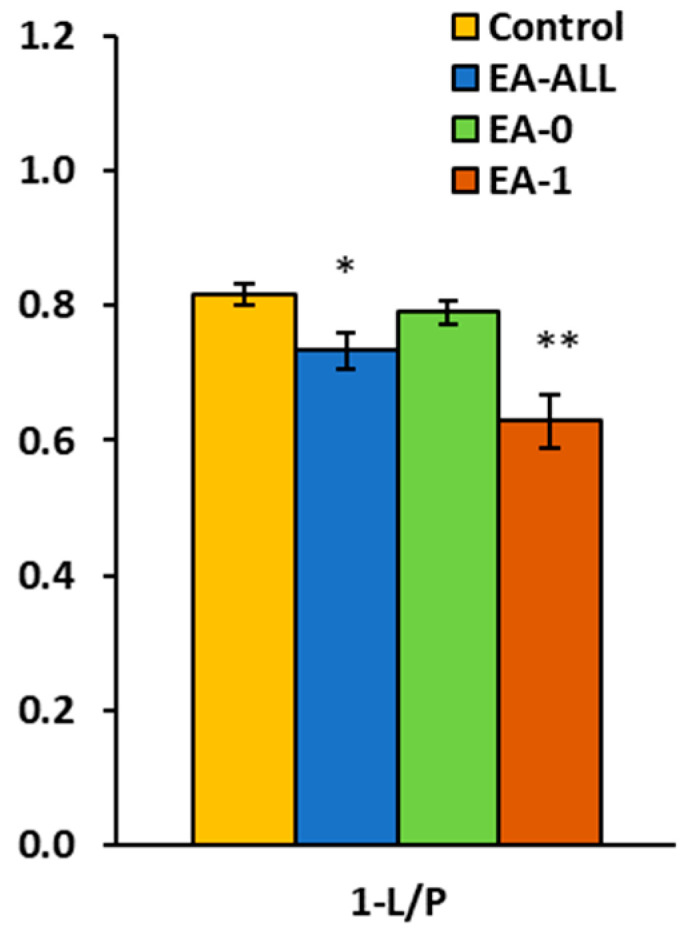
Protocol RP1: *P-L* OXPHOS efficiency in platelet mitochondria of patients with Ebstein anomaly (1-L/P). * *p* < 0.05; ** *p* < 0.01.

This parameter was significantly decreased (*p* = 0.014) in the EA-All group, reaching 90% of the control group value. In the EA-0 group, this parameter reached 97% of control values; in the EA-1 group, this was 77% (*p* = 0.006). These results indicate significantly lower efficiency of OXPHOS associated with mitochondrial CI-linked respiration (Figure 2, Table 2).

### 2.5. Platelet Mitochondrial Respiration and Fatty Acid Oxidation in EA Patients by Protocol RP2

**Figure 3:**
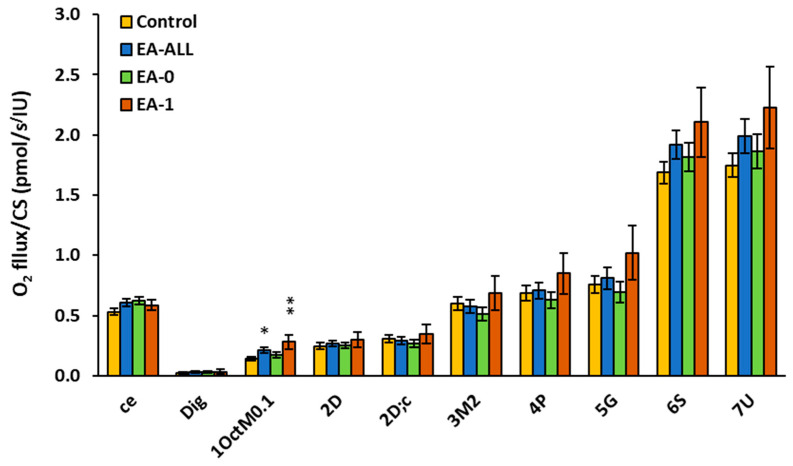
Platelet mitochondrial respiration and fatty acid oxidation is increased in EA patients by protocol RP2. Legend: Control group (C), group of all patients with Ebstein anomaly (EA-All), EA patients with non-failing heart (EA-0) and EA patients with failing heart (EA-1). The respiration was measured in freshly isolated platelets following substrate–uncoupler–inhibitor titration (SUIT) reference protocol RP2 [29] and evaluated as O_2_ flux per mitochondrial marker citrate synthase activity. The graph shows mean ± SEM of the respiratory capacities after the titration step indicated on the x-axis: ce—oxygen consumption in intact platelets; Dig—digitonin—residual oxygen consumption after platelet permeabilization; 1OctM0.1—octanoylcarnitine with 0.1 mM malate saturating FAO; 2D—ADP addition—the respiration associated with ATP production (FAO—linked OXPHOS); 2D;c—cytochrome c; 3M2—malate saturating CI-linked respiration; 4P—pyruvate addition; 5G—glutamate addition; 6S—succinate addition; 7U—uncoupler addition. * *p* < 0.05; ** *p* < 0.01.

The results of platelet mitochondrial respiration and fatty acid oxidation by protocol RP2 in EA patients are presented in Figure 3 and Table 2. In intact platelets, oxygen consumption (*ce*) was slightly increased in the EA-All group vs. the control group (to 115%). In the EA-0 group, *ce* respiration was increased to 118% vs. control data; in the EA-1 group, this was 111%. After the permeabilization of platelets with digitonin (Dig) and supplementation with the fatty acid oxidation (FAO)-linked substrate octanoylcarnitine plus 0.1 mM malate (1OctM0.1), the respiration in the EA-All group was significantly increased (*p* = 0.03) to 149%; in the EA–0 group, it was increased to 122%; and in the EA–1 group, it was increased (*p* = 0.006) to 197% vs. control data. FAO-linked OXPHOS respiration capacity after ADP addition (2D) associated with ATP production was not changed in EA-All patients vs. control data. Different trends in platelet mitochondrial respiration in EA groups were found after cytochrome *c* (2D;c) addition: in the EA-0 group, respiration reached 86% vs. control data; in the EA-1 group, this parameter increased to 113% of the control value. A similar trend in mitochondrial respiration was found after substrate malate 3M2 addition. In the EA–0 group, respiration reached 85%; in the EA-1 group, it was stimulated to 115%. After the addition of substrate pyruvate (4P), platelet mitochondrial respiration in the EA–0 group was slightly decreased to 91%; in the EA–1 group, it was increased to 123%. After the addition of substrate glutamate (5G), platelet mitochondrial respiration in the EA–0 group reached 92%; in the EA–1 group, it was increased to 135% vs. control. After the addition of substrate succinate (6S), and after the addition of uncoupler (7U), platelet mitochondrial oxygen consumption was slightly stimulated in all groups of EA patients in comparison with control values. These differences did not reach statistical significance. These platelet mitochondrial respiratory changes may indicate different metabolic cells pathways for the adaptation of non-failing (EA-0 group of patients) and failing hearts (EA-1 group of patients) [12] (Table 3, Figure 3).

### 2.6. The Efficiency of FAO-Linked OXPHOS in EA Patients by Protocol RP2

**Figure 4:**
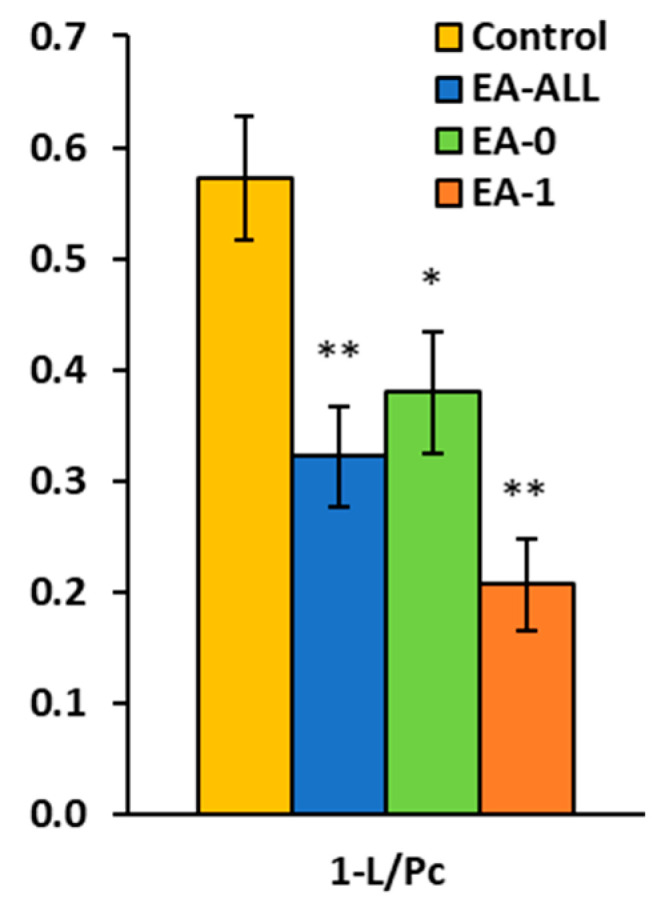
P-L coupling efficiency of FAO-linked OXPHOS in EA patients by protocol RP2. * *p* < 0.05; ** *p* < 0.01.

P-L coupling efficiency of FAO and OXPHOS (1-L/P*c*) was statistically significantly reduced in EA-All patients (*p* = 0.003) to 56% of control values, in the EA-0 group to 66% (*p* = 0.038) of control values, and in the EA-1 group to 36% (*p* = 0.005) of control group values. These results indicate the uncoupling of mitochondrial oxidation and phosphorylation (Table 3, Figure 4).

### 2.7. Endogenous Antioxidants and TBARS in Patients with Ebstein Anomaly

**Figure 5:**
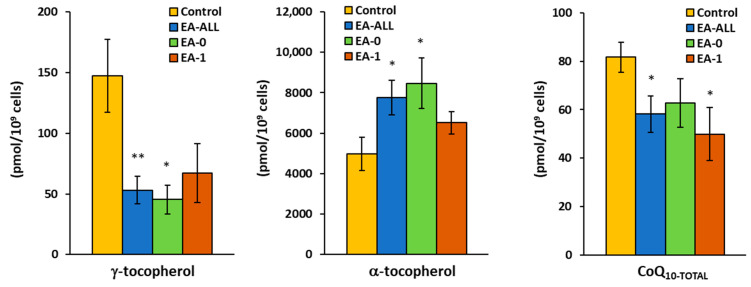
γ-tocopherol, α-tocopherol, and coenzyme Q_10-TOTAL_ concentration in platelets of EA patients (see Table 4). Legend: CoQ_10-TOTAL_—coenzyme Q_10-TOTAL_ (ubiquinol + ubiquinone); values are in pmol/10^9^ cells: * *p* < 0.05; ** *p* < 0.01.

**Figure 6:**
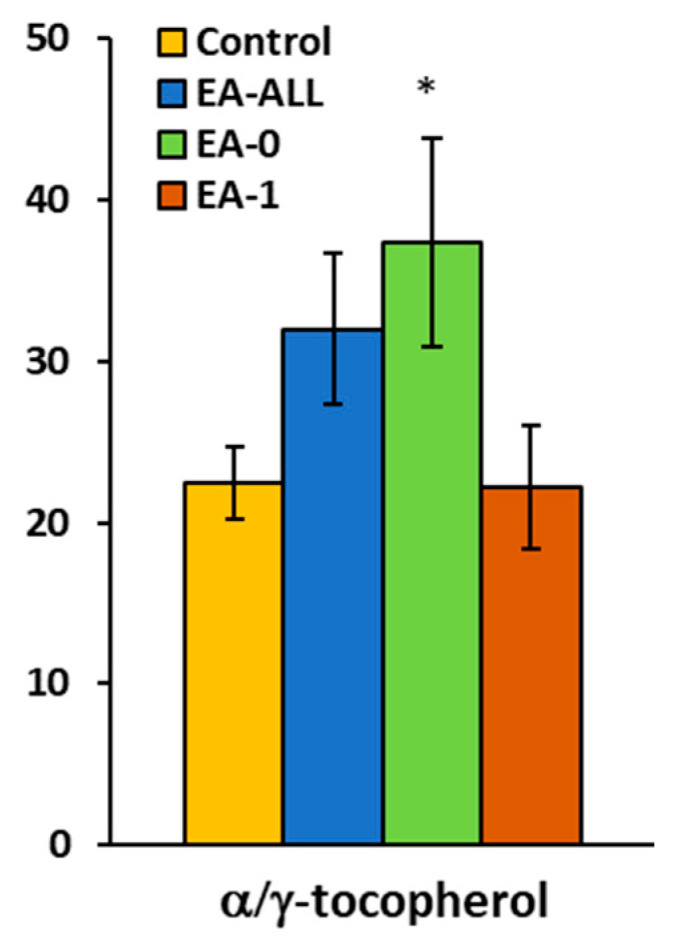
The alpha/gamma-tocopherol ratio in plasma in patients with Ebstein anomaly was increased (see Table 4). * *p* < 0.05.

Platelet γ-tocopherol concentration was significantly reduced in all groups of patients: in EA-All, pts reached 35% of control values (*p* = 0.003); in EA-0, patients reached 29% (*p* = 0.009) of control values; and in EA-1, patients reached 44% (*p* = 0.11) of control group values (Figure 5, Table 4). Platelet α-tocopherol in EA-All patients increased to 168% (*p* = 0.009); in EA-0 patients, it increased to 183% (*p* = 0.010); and in EA-1 patients, it increased to 141% (*p* = 0.14). In plasma of EA-1 patients, α-tocopherol reached 85% of control group values (Table 4). The CoQ_10-TOTAL_ (ubiquinol + ubiquinone) concentration in platelets of EA-All patients was decreased to 69% (*p* = 0.009); in EA-0 patients, it was decreased to 74% (*p* = 0.056); and in EA-1 patients, it was reduced to 59%, (*p* = 0.011) (Figure 5, Table 4). CoQ_10-TOTAL_ concentration in plasma was decreased in EA-pts to 83–85% of control group values (Table 4). Plasma lipoperoxidation (TBARS) was not different between EA patients and the control. The αT/γT ratio in the EA-All group increased to 126%; in EA-0, it increased to 147% (*p* = 0.054); and in EA-1, it reached 87% of control group values (Figure 6, Table 4).

The non-failing heart group of EA-0 patients and failing heart group of EA-1 patients differ by the ratio of α-tocopherol/γ-tocopherol (*p* = 0.13).

### 2.8. The Correlations Between Platelet Concentration of CoQ_10_ and Respiratory Parameters of RP1 and RP2 in Patients with Ebstein Anomaly: EA-0, EA-1 Groups

**Figure 7:**
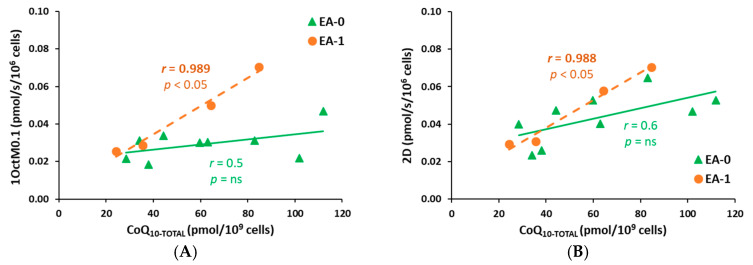

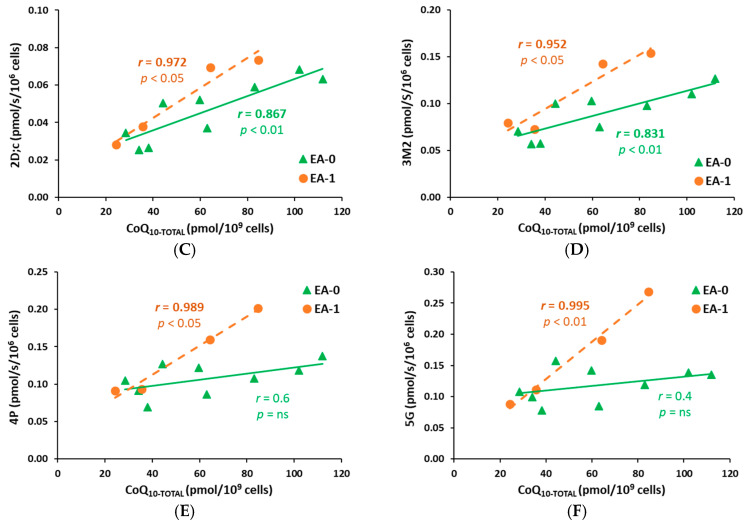
(**A**–**F**) The correlations between platelet CoQ_10_ concentration and respiratory parameters of RP2 in EA patients. Legend: *r*—Pearson correlation coefficient, *p*—statistical significance of the correlation.

The correlations between CoQ_10_ concentration in platelets and the parameters of mitochondrial respiration in platelets were statistically non-significant in the RP1 protocol. In case of the RP2 protocol, some of the parameters of mitochondrial respiration in platelets were significantly correlated with CoQ_10_ concentration in platelets (Figure 7A–F). The correlations were more significant in the EA-1 group of patients with a failing heart, suggesting a stronger dependence of mitochondrial respiration on intracellular CoQ_10_ level in this group of patients. However, interpretation is limited by a small number of patients in this group.

## 3. Discussion

Cardiovascular diseases are associated with dysfunction of mitochondrial dynamics and bioenergetics, oxidative stress, and mitochondrial DNA (mtDNA) alterations. As the primary energy source and a major site of ROS production, mitochondria play a central role in cardiac health [2,6,7]. Under pathological conditions, cardiomyocytes undergo metabolic adaptation to compensate for energy deficits. This process is associated with reduced fatty acid import into cardiomyocytes and decreased ATP production. As the disease progresses toward clinical heart failure, cardiomyocytes obtain energy from glucose and ketone bodies, with a lower yield of ATP production. This reduced ATP production further impairs myocardial contractility [30].

Mitochondrial dynamics, including mitophagy (the removal of damaged mitochondria) and biogenesis (the creation of new ones), are significantly altered in the pathological myocardium [12]. These changes lead to reduced FAO, impaired membrane potential, and electron transport chain dysfunction [11,20]. Such pathobiochemical shifts contribute directly to heart failure [31,32]. Furthemore, uncontrolled ROS production and energy deficits eventually lead to organ failure [4,33]. While moderate oxidative stress can stimulate mitochondrial biogenesis (*mitohormesis*) [34,35], chronic ROS elevation triggers the opening of the mitochondrial permeability transition pore (MPTP), loss of membrane potential, and cytochrome *c* release from mitochondria into the cytoplasm, which triggers apoptosis. In pathological conditions of the myocardium, myocardial metabolism is reprogrammed; glycolysis is preferred instead of fatty acid oxidation [4].

A prototype model of RV dysfunction driven by chronic volume overload is represented by EA. This rare CHD is characterized by the pathognomonic apical displacement of tricuspid valve leaflets. The resulting severe tricuspid regurgitation leads to progressive RV dilation and systolic dysfunction. While surgical repair is increasingly performed at earlier stages to prevent disease progression, the optimal timing for intervention before irreversible damage occurs remains elusive. This underscores the critical need for reliable biomarkers to guide clinical decision-making and optimize the ideal timing for surgery. Consequently, EA provides a unique clinical framework for investigating the bioenergetic and metabolic shifts that underlie myocardial failure.

In this study, we used isolated platelets from circulating blood to assess mitochondrial bioenergetic function in patients with EA. Platelets are easily available anucleate cells from peripheral blood, containing 5–8 metabolically active mitochondria; they live for 7–10 days. They arise from megakaryocytes in the bone marrow and are released into the circulation. Platelet mitochondria play an important role in myocardial function [36]. Mitochondria released from platelets (extracellular mitochondria) are major factors in the metabolic remodeling of mesenchymal stem cells [37,38].

### 3.1. Platelet Mitochondrial Respiration in EA Patients (Protocol RP1)

Our results showed that mitochondrial CI-linked LEAK respiration significantly increased in both groups of EA patients compared to controls. The differential increase in CI-linked LEAK respiration with substrates pyruvate and malate may reflect the clinical severity of the diseases. In EA-0 patients, this likely represents a transient state of myocardial adaptation and metabolic reprogramming. Conversely, in EA-1 patients, this may reflect extensive dysregulation involving fatty acid, amino acid, and lipid metabolism, as well as the Krebs cycle. Increased mitochondrial respiration may result from electron accumulation at the NADH site (Complex I) of the mitochondrial respiratory chain. Electrons change direction, bind to oxygen, and form hydroxyl radicals, leading to oxidative stress. Interestingly, we found only a slight increase in lipid peroxidation in the plasma of EA patients (Table 4), which may be related to the relatively small sample size of EA patients.

Metabolic reprogramming may shift substrate preferences, leading to increased oxygen consumption at the CI-NADH complex. Similar increases in mitochondrial respiratory capacity in PBMCs have been suggested as prognostic markers for heart failure risk in single-ventricle congenital heart diseases [17]. Despite these changes, ATP production in platelet mitochondria in EA patients remained similar to controls. In agreement with authors [39,40], we suggest that mitochondria of the heart RV adapt to high energy demands by utilizing less efficient pathways, such as the metabolism of carbohydrates, ketone bodies, or amino acids.

There was a minor difference in several other parameters of platelet mitochondrial respiration between the groups of patients with EA-0 and EA-1. Increased platelet LEAK respiration in EA patients with RV dysfunction in heart failure is part of a broader metabolic reprogramming that is a response to chronic stress and altered hemodynamic conditions in the body. The increase in respiration after the addition of cytochrome *c*, a mobile component of the respiratory chain, was small and similar in all groups (Figure 1, Table 2). Cytochrome *c* can be released from the intermembrane space via the voltage-dependent anion channel (VDAC) of the outer mitochondrial membrane [41]. OXPHOS efficiency (1-L/P) coupled to CI was reduced in the EA-All and EA-1 groups, indicating a partial uncoupling of oxidation from phosphorylation. Our findings demonstrate increased CI-linked LEAK mitochondrial respiration and decreased OXPHOS efficiency in the platelets of EA patients compared to controls.

### 3.2. Platelet Mitochondrial Respiration in EA Patients (Protocol RP2)

Mitochondrial respiration results according to the RP2 protocol are summarized in Figure 3 and Figure 4 and Table 3. This study focused on fatty acid oxidation (FAO) using the RP2 protocol, yielding results consistent with the RP1 findings. Following the addition of octanoylcarnitine and malate, LEAK mitochondrial respiration was increased in both groups of EA patients compared to controls.

Distinct differences in FAO were noted between the EA-0 and EA-1 groups after the addition of CI-linked substrates (malate, pyruvate, glutamate). While EA-0 patients showed a lower respiration compared to controls, EA-1 patients exhibited higher oxygen consumption. A similar trend was observed in ET capacity for FAO&CI&CII-linked substrates. These differences highlight a deterioration in FAO and CI-linked OXPHOS capacity specifically in EA-1 patients; however, the differences were not statistically significant.

OXPHOS efficiency, expressed as 1-L/Pc (linked to FAO), was significantly reduced in both EA groups, representing a profound uncoupling of oxidation from phosphorylation. Another potential mechanism for mitochondrial bioenergy changes could be carnitine cycle dysfunction. Authors [32] showed dysregulation of this cycle associated with reduced CPT I and CPT II enzyme activities in single-ventricle hearts. In the absence of sufficient fatty acids, metabolism is reprogrammed toward less efficient sources, such as glycolysis [12,32].

In both protocols, the only statistically significant difference between EA patients and the control group was elevated CI-linked LEAK respiration. This elevation was significant in the EA-ALL and EA-1 groups, but not in the EA-0 group. We suggest that the determination of OXPHOS characteristics in platelets could serve as systemic metabolic markers for EA. In conclusion, metabolic reprogramming in EA patients involves a metabolic shift from fatty acid oxidation toward increased glucose and potentially ketone body metabolism, alongside altered carnitine and Krebs cycle activity. While these maladaptive changes aim to alleviate electron accumulation and maintain redox balance, they ultimately contribute to the progression of myocardial RV dysfunction [42].

### 3.3. Endogenous Antioxidants and TBARS in Patients with Ebstein Anomaly

Coenzyme Q_10_ (CoQ_10_) is an integral component of mitochondrial respiratory chain supercomplex (CI + CIII + CIV), which facilitates efficient electron transfer to Complex II and Complex III. One pool of CoQ_10_ is sequestered within this supercomplex for NADH oxidation at Complex I, while another pool remains free within the membrane to serve Complex II, electron transferring flavoprotein, glycerophosphate dehydrogenase, choline dehydrogenase and dihydro-orotate dehydrogenase. Under conditions of extreme electron pressure on CI and a deficiency of CoQ_10_, reverse electron transport (RET) toward NADH-CI occurs. This process induces the formation of superoxide radicals, leading to the degradation of mitochondrial structures [43].

In EA patients, increased CI-linked LEAK respiration, associated with a deficiency in endogenous CoQ_10_ synthesis, may be caused by several pathobiochemical mechanisms.

CoQ_10_ deficiency induces reverse electron transport (RET) from the CoQ_10_ pool back toward Complex I (CI). The resulting accumulation of electrons at CI leads to the partial reduction of oxygen, serving as a primary source of superoxide radical production and initiating a cascade of oxidative stress. Electron accumulation and ROS formation at a high electrochemical gradient may also be related to passive proton leakage from the inner mitochondrial membrane (IMM) back into the matrix, instead of toward Complex V—ATP synthase. Increased LEAK means that IMM becomes more permeable to protons that do not pass through ATP synthase.

CoQ_10_ is part of the supercomplex CI + CIII + CIV (respirasome); it supports the stability of the supercomplex. CoQ_10_ deficiency disrupts the architecture and efficiency of mitochondria, leading to the dissociation of the supercomplex, and less stable complexes are formed. CoQ_10_ deficiency causes the breakdown of respirasomes and degradation of CI, leading to an energic collapse of the cell [44,45,46]. It is believed that CoQ_10_ deficiency reduces the activity of Sirtuin 1 and Sirtuin 3, key enzymes and deacylases dependent on NAD+, which regulate cellular metabolism [47].

In EA, chronic overload and structural changes in the RV heart occur. The result is oxidative stress and damage to the integrity of the IMM. The leakage of protons from mitochondria through the IMM provides protection against oxidative stress [48]. With increased mitochondrial respiration, the energy of the proton gradient is used to generate heat, instead of ATP production, which is reduced, and an uncoupling effect occurs, the disconnection of oxidation from phosphorylation. In Ebstein anomaly, the RV is mechanically overloaded and inefficient. The heart needs more energy than the mitochondria can produce, and ATP breaks down in the ADP, which accumulates in the cell and is an endogenous signal of energy crisis. Furthemore, increased ROS production further destabilizes Complex I, leading to its degradation and the subsequent accumulation of NADH. The resulting elevation in the NADH/NAD^+^ ratio is indicative of reductive stress [40,43,49].

Endogenous platelet CoQ_10_ levels were significantly diminished in EA patients compared to controls. This deficit likely stems from the varying degrees of myocardial dysfunction. CoQ_10_ deficiency prevents efficient electron transfer to CII and CIII, inducing RET toward CI. This may be the primary mechanism for the increased mitochondrial respiration observed at the Complex I site in patients with EA. The resulting reductive stress, a signal of oxidative stress, triggers the opening of the mitochondrial permeability transition pore (MPTP) and the loss of membrane potential [4,43]. As a central regulator of metabolic reprogramming [43], reductive stress modulates cellular metabolism, energy production, and stress responses [50,51]. While this reprogramming is a compensatory mechanism aimed at restoring redox homeostasis [42], exceeding this adaptive capacity ultimately triggers apoptosis.

Other platelet antioxidants in EA patients were also affected. The concentration of endogenous γ-tocopherol in platelets was reduced in both groups of EA patients (Table 4). One possible cause for this deficiency is the stimulation of CYP450 activity by high doses of α-tocopherol, which may accelerate the degradation of γ-tocopherol through a competitive mechanism. Conversely, platelet α-tocopherol concentrations were elevated in both EA groups. This significant increase in α-tocopherol, coupled with γ-tocopherol deficiency, may impair the overall antioxidant and anti-inflammatory capacity and also be indicative of impaired liver function [52]. Furthemore, dysfunctional mitochondria can subsequently activate platelets, increasing the risk of thromboembolic events.

We hypothesize that the increased LEAK respiration in EA patients is a compensatory mechanism for the high energy demands of the RV myocardium. In EA-0, this may represent a successful cellular adaptation. However, in EA-1 patients, these findings likely signal maladaptive reprogramming and an increased risk of heart failure, driven by electron accumulation and a high NADH/NAD^+^ ratio in the presence of CoQ_10_ deficiency [43]. Ultimately, these alterations in platelet mitochondrial bioenergetics may reflect systemic mitochondrial dysfunction across other tissues.

## 4. Materials and Methods

### 4.1. Subjects

Patients with Ebstein anomaly (*n* = 14) and healthy volunteers (*n* = 18) were included. According to the 2020 recommendations of the European Society of Cardiology for congenital heart defects in adulthood [28], EA patients were divided into two groups: EA-0 (severe tricuspid regurgitation with difficulties, non-failing heart), EA-1 (progressive dysfunction or dilatation of the right ventricle, failing heart). For the control group, see part 2.1.

### 4.2. Metabolic Parameters of Patients with Ebstein Anomaly

All measured parameters are given with the reference values in Table 1.

### 4.3. Isolation of Human Platelets

Ten ml of venous blood was collected by venipuncture in K3EDTA (tripotassium ethylenediaminetetraacetic acid) tubes. Platelets (PLT) were isolated from the whole blood by centrifugation of the tubes at room temperature at 200× *g* for 10 min without braking using a swing-out rotor. Platelet-rich plasma (PRP) was transferred into a new plastic tube and mixed with 100 mM EGTA (ethylene glycol-bis (2-aminoethyl ether)-*N*,*N*,*N*’,*N*’-tetraacetic acid) to a final concentration 10 mmol/L EGTA. The tubes were centrifuged at 1200× *g* for 10 min, then the pellet was washed with 4 mL of DPBS (Dulbecco’s phosphate-buffered saline) plus 10 mM EGTA and centrifuged at 1200× *g* for 5 min. The pellet was resuspended in 0.4 mL of the same solution, and platelet suspension was counted (10 times diluted) by using the hematological analyzer Mindray BC-6200 (Mindray, Shenzhen, China) [53].

### 4.4. High-Resolution Respirometry Method for Mitochondrial Bioenergetic Function by SUIT Protocols RP1 and RP2

For mitochondrial respirometry in freshly isolated platelets, the high-resolution respirometry (HRR) method was used. Platelets (250 × 10^6^ cells) for respirometric analysis were added into a 2 mL chamber of an O2k-Respirometer (Oroboros Instruments, Innsbruck, Austria) to the medium MiR05 with 20 mM creatine at 37 °C under continuous stirring at 750 rpm. The data were collected with DatLab software (version 8.0, Oroboros Instruments GmbH, Austria) [54,55].

**Figure 8:**
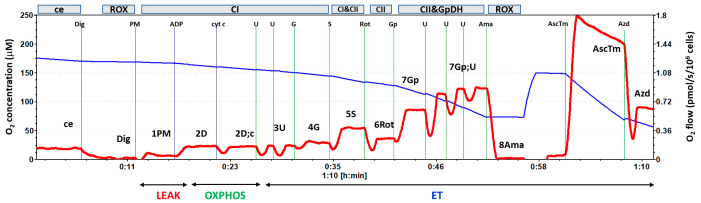
Record of platelet mitochondrial respiration by HRR method using protocol RP1 for OXPHOS. Legend: The trace from the measurement of platelet mitochondrial respiration following SUIT reference protocol RP1 [29,57]. The blue line represents oxygen concentration (μM), and the red trace represents oxygen consumption as flow per cells (pmol O_2_/s/10^6^ cells). The protocol includes the following titration steps: ce—cells; Dig—Digitonin; PM—pyruvate + malate; ADP—ADP addition for ATP production; cyt c—cytochrome c; U—the uncoupler FCCP; G—glutamate; S—succinate; Rot—rotenone (CI inhibitor); Gp—glycerophosphate; U—the uncoupler FCCP; Ama—antimycin A; AscTm—artificial substrates of CIV; Azd—sodium azide (CIII inhibitor). All substrates were titrated in kinetically saturating concentrations; uncoupler FCCP was titrated in the optimum concentration for the evaluation of maximum O_2_ flow at a given titration step. The evaluated respiratory capacities are marked according to the titration steps in the reference protocol RP1 and correspond to the following respiratory states: ce—routine respiration of intact platelets; Dig—residual oxygen consumption (ROX) after permeabilization with digitonin; 1PM—LEAK respiration with CI-linked substrates pyruvate + malate; 2D—CI-linked OXPHOS capacity; 2D;c—CI-linked OXPHOS capacity after addition of cytochrome *c*; 3U—CI-linked electron transfer (ET) capacity with pyruvate + malate; 4G—CI-linked ET capacity with pyruvate + malate + glutamate; 5S—CI&II-linked ET capacity; 6Rot—CII-linked ET capacity; 7Gp;U—CII&GpDH-linked ET capacity; 8Ama—residual oxygen consumption; CIV—the activity of CIV with artificial substrates TMPD + ascorbate [58,59].

The substrate–uncoupler–inhibitor titration (SUIT) protocol (RP1) was used for platelet mitochondrial respiration and energy production by OXPHOS. The following parameters of respiration were measured: ce—respiration of intact platelets; Digitonin was used to permeabilize the cell membrane (Dig). Mitochondrial Complex I (CI)-linked LEAK respiration was measured after subsequent addition of CI-linked substrates: 5 mM pyruvate and 2 mM malate (1PM). CI-linked oxidative phosphorylation (OXPHOS) capacity (2D), the parameter of mitochondrial respiration associated with ATP production, was determined after the addition of saturating ADP concentration (1 mM). The addition of cytochrome *c* (10 μM) was used for testing the integrity of the outer mitochondrial membrane (2D;c). Maximum oxidative capacity with CI-linked substrates (CI-linked ET capacity) (3U) was evaluated after the titration of the uncoupler (FCCP, 0.5 μM steps) to maximum respiration, CI-linked electron transfer (ET) capacity after the addition of 10 mM glutamate (4G) and CI&CII-linked ET capacity after the addition of Complex II (CII)-linked substrate (10 mM) were determined (5S). The protocol continued with the addition of an inhibitor of CI, rotenone (0.75 μM) (6Rot), allowing the determination of CII-linked ET capacity [29,56], 10 mM glycerophosphate (7Gp), additional uncoupler titration until maximum respiration (7Gp;U), and 2.5 μM antimycin A (8Ama), an inhibitor of CIII inhibiting mitochondrial pathway-linked respiration. Subsequently, the oxygen concentration in the chamber was increased to 150 mM, and artificial substrates of CIV 0.5 mM *N*,*N*,*N*’,*N*’-Tetramethyl-p-phenylenediamine dihydrochloride (TMPD) and 2 mM ascorbate were added (AscTm). After 5 min, CIV inhibitor sodium azide (100 mM) was added (Azd). CIV activity was evaluated based on difference in O_2_ flux with TMPD + ascorbate and O_2_ flux after the addition of sodium azide. For the evaluation of mitochondrial respiration, the residual oxygen consumption (ROX) after 8Ama or Dig (the lower value) was subtracted from respiration in all steps of the protocol. The record of the measurement is in Figure 8.

**Figure 9:**
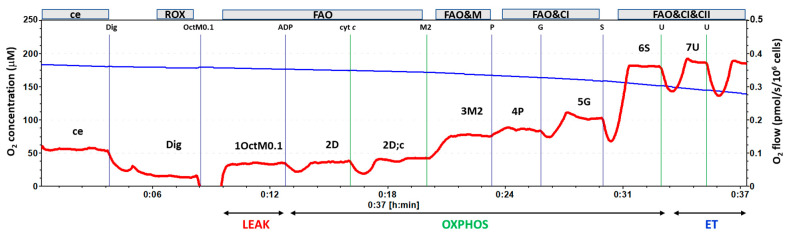
Record of platelet mitochondrial respiration by HRR method using SUIT protocol RP2 for FAO. Legend: The blue line represents oxygen concentration (μM), and the red trace represents oxygen consumption as flow per cells (pmol O2/s/10^6^ cells). A total of 250 × 10^6^ platelets were used in a 2 mL chamber of an O2k-respirometer. The respiration was measured in mitochondrial respiration medium MiR05 with 20 mM creatine at 37C under continuous stirring at 750 rpm. The protocol includes the following titration steps: Dig—digitonin; 1OctM0.1—octanoylcarnitine plus 0.1 mM malate saturating FAO; 2D—ADP; 2D;c—cytochrome *c*; 3M2—2 mM malate saturating CI-linked respiration in the presence of malate; 4P—pyruvate, 5G—glutamate; 6S—succinate; 7U—uncoupler FCCP titration until maximum.

The substrate–uncoupler–inhibitor titration (SUIT) protocol (RP2) was used for evaluation of the (FAO)-pathway, avoiding FAO overestimation in the presence of anaplerotic pathways. In this protocol, after stabilization at routine respiration of intact cells (ce), digitonin (0.20 μg/10^6^ cells) was used for permeabilization of the cell membrane (Dig). Then, octanoylcarnitine (0.5 mM) and malate (0.1 mM) (OctM) were added (in concentrations saturating FAO) to induce fatty acid oxidation; 1 mM ADP-Mg (0.6 mol MgCl_2_/mol ADP) (2D) was added to stimulate OXPHOS with FAO-pathway substrates; and cytochrome *c* (10 μM) was added for testing the integrity of the outer mitochondrial membrane (2D;c). The increase in respiration after cytochrome *c* would indicate impaired integrity of the outer mitochondrial membrane. Next, 2 mM malate (M2) was added to test for the presence of malic enzyme or other anaplerotic pathways (together FAO&M-pathway). Malate in 2 mM concentration is saturating for CI-linked respiration in the presence of pyruvate. The addition of 2 mM malate before pyruvate is the test for the presence of malic enzyme or other anaplerotic pathways by which pyruvate is formed from malate, and, therefore, malate stimulates CI-linked oxygen consumption similarly to the presence of both pyruvate and malate. Subsequently, 5 mM pyruvate (P) was added to support the CI-pathway (together FAO&CI-pathway). As can be seen from the trace (Figure 9), relatively high activity of malic enzyme or other anaplerotic pathways is present in platelets, as the addition of pyruvate after 2 mM malate causes only a small increase in respiration. Next, 10 mM glutamate (G) was added to complete the CI-pathway; then, 10 mM succinate (S) was added to support convergent electron flow from the FAO&CI&II-pathway, and uncoupler FCCP was titrated to reach maximal ET capacity with FAO&CI&II-linked substrates.

### 4.5. Citrate Synthase Activity

The activity of citrate synthase as a mitochondrial marker was evaluated by the spectrophotometric method [60,61]. All assays were conducted with a UV–Visible spectrophotometer Biochrom 4060 (Biochrom Ltd., Cambridge, UK) using 0.3 mL sample cuvettes at 25 °C. The reaction mixture contained 100 mM Tris-HCl pH = 8.1, 101 mM 5,5′-dithiobis (2-nitrobenzoic acid), 0.25% Triton X-100, and 0.3 mM acetyl-CoA. After the addition of platelets from thawed suspension (15 × 10^6^ cells/measurement), the reaction was initiated by the addition of 0.5 mM oxaloacetate and followed at 412 nm for 2 min. The activity of CS (in μmol/min/10^6^ cells = IU) was calculated from the rate of concentration change using the extinction coefficient of thionitrobenzoic acid 13.6/mM/cm. All parameters of oxygen consumption were evaluated as *JO2/CS* (pmol/s/IU) [58].

### 4.6. Coenzyme Q_10,_ γ-Tocopherol, α-Tocopherol Determination in Platelets

Isolated human platelets (150–250 × 10^6^ cells) were disintegrated with 500 μL of cold methanol [62]. Oxidation of ubiquinol to ubiquinone was performed with 1,4-benzoquinone. CoQ_10-TOTAL_ (ubiquinol + ubiquinone) in isolated platelets was determined using the HPLC method with UV detection at 275 nm; γ-tocopherol and α-tocopherol were detected at 290 nm in the same measurement using external standards (Sigma, St. Louis, MO, USA) [63,64,65]. Data were collected and processed with a CSW32 chromatographic station (DataApex).

### 4.7. Determination of TBARS in Plasma

Thiobarbituric acid reactive substances (TBARSs) as a parameter of oxidative stress were determined by the spectrophotometric method [66]. Plasma samples (0.5 mL) were mixed with 0.15 mL ice-cold 76% trichloroacetic acid (TCA), 0.35 mL 1.07% thiobarbituric acid and 1 mL of H_2_O. Samples were incubated at 100 °C for 30 min and after cooling down, 0.5 mL of 90% TCA was added. After centrifugation at 2200× *g* for 15min, the absorbance of supernatant was measured at 532 nm with a UV–visible spectrophotometer (Biochrom 4060), and the concentration was calculated in μmol/L.

### 4.8. Data Analysis

The data were statistically evaluated with GraphPad Prism 6 for Windows (GraphPad Software, Boston, MA, USA). Unpaired Student’s *t*-tests were applied to evaluate the differences between the patient groups and the control group. The results in graphs and tables are expressed as mean ± standard error of mean (SEM). In case of respiration, data show ROX-corrected fluxes. Values of *p* < 0.05 were considered statistically significant. The values of the measured parameters were also evaluated in % compared to the control values, which were considered 100%.

## 5. Conclusions

Alterations in mitochondrial bioenergetics and reduced endogenous CoQ_10_ levels appear to be key drivers of mitochondrial reprogramming in patients with Ebstein anomaly. This reprogramming favors ROS production in NADH-CI-linked respiration and suggests a metabolic shift toward glycolysis over fatty acid oxidation (FAO). Based on our findings, we hypothesize that the stimulation of platelet LEAK mitochondrial respiration, as observed in both RP1 and RP2 protocols, may serve as a significant biomarker for predicting progression of RV. Furthermore, the analysis of platelet mitochondrial bioenergetics offers valuable insights into the pathobiochemical mechanisms of EA. Determination of platelet mitochondrial bioenergetics by high-resolution respirometry and the assessment of endogenous CoQ_10_ levels provide a promising approach facilitating personalized medicine and potentially refining surgical timing and optimizing the long-term management of patients with EA. Translating these findings into clinical practice requires further validation in larger perspective cohorts to establish precise diagnostic cut-off values. Integrating these bioenergetic markers with established indicators, such as NT-proBNP, echocardiography, and cardiac magnetic resonance imaging, will be essential to confirm their additive predictive value and ensure their effective implementation in personalized management strategies for EA patients.

### Limitation of the Study

There was a relatively smaller number of patients with Ebstein anomaly (women (*n* = 12), men (*n* = 2)). EA is a rare CHD, and our cohort of 14 patients represents all patients in our national registry fulfilling the inclusion criteria. We agree with the reviewer’s comments. Several results in a small number of patients also indicate a trend toward statistical significance; the impact of the results on gender cannot be assessed from the small number of patients. We consider these results to be pilot and preliminary. Additional studies are needed.

## Figures and Tables

**Table 1:** Metabolic Parameters in Patients with Ebstein Anomaly. Legend: Groups: n = number of patients; EA-All = 14; EA-0 = 9; EA-1 = 5; M = male; F = female. All parameters are in references values; significant differences were found between EA-0 and EA-1 groups in Bilirubin total (** *p* = 0.0037) and Hemoglobin (* *p* = 0.02).

Parameters	Ref.val. M	Ref.val. F	EA-All pts	EA-0 pts	EA-1 pts
AST (µkat/L)	M: <0.85	F: <0.60	0.429 ± 0.047	0.426 ± 0.071	0.436 ± 0.049
ALT (µkat/L)	M: 0.10–0.80	F: 0.10–0.60	0.339 ± 0.044	0.300 ± 0.06	0.408 ± 0.057
GMT (µkat/L)	M: <0.82–0.92	F: <0.53–0.63	0.559 ± 0.096	0.573 ± 0.132	0.534 ± 0.146
ALP (µkat/L)	M: 0.90–2.20	F: 0.7–2.1	1.044 ± 0.073	0.967 ± 0.083	1.182 ± 0.126
Bilirubin total (µmol/L)	3.4–17.1		10.82 ± 1.72	7.5 ± 1.3	16.8 ± 2.7 **
Cholesterol total (mmol/L)	<5.00		4.30 ± 0.25	4.29 ± 0.24	4.32 ± 0.60
HDL-cholesterol (mmol/L)	M: 1.08–2.20	F: 1.20–2.05	1.60 ± 0.12	1.72 ± 0.16	1.39 ± 0.16
LDL-cholesterol (mmol/L)	<3.0		2.50 ± 0.24	2.24 ± 0.22	2.95 ± 0.50
TAG (mmol/L)	<1.7–2.2		1.47 ± 0.15	1.47 ± 0.17	1.46 ± 0.32
Glucose (mmol/L)	3.3–5.6		5.10 ± 0.17	4.89 ± 0.19	5.47 ± 0.31
Creatinine (mmol/L)	M: 64–104	F: 49–90	72.8 ± 3.1	72.6 ± 4.7	73.2 ± 2.3
Urea (mmol/L)	2.8–8.0		5.69 ± 0.29	5.64 ± 0.37	5.78 ± 0.55
Uric acid (µmol/L)	M: 200–430	F: 140–360	276.9 ± 25.9	244.0 ± 22.6	336.2 ± 53.6
Albumin (g/L)	M: 60–85	F: 35–52	44.5 ± 0.9	45.3 ± 1.4	43.0 ± 0.8
Hemoglobin (g/L)	M: 135–175	F: 120–155	141.6 ± 5.9	131.8 ± 5.1	159.2 ± 10.3 *

**Table 2:** Platelet mitochondrial respiration and OXPHOS in patients with Ebstein anomaly by protocol RP1 (O_2_ flux/CS (pmol/s/IU)). Legend: see Figure 1.

Parameters	Groups	Control	EA-All pts	EA-0 pts	EA-1 pts
	N	17	14	9	5
**ce**	Mean ± SEM	0.583 ± 0.028	0.609 ± 0.034	0.626 ± 0.046	0.577 ± 0.048
	*p*-value		0.56	0.40	0.92
	% of control		104%	107%	99%
**Dig**	Mean ± SEM	0.016 ± 0.007	0.010 ± 0.004	0.008 ± 0.006	0.014 ± 0.005
	*p*-value		0.48	0.43	0.89
	% of control		63%	48%	89%
**1PM**	Mean ± SEM	0.134 ± 0.017	0.227 ± 0.038	0.160 ± 0.019	0.346 ± 0.081
	*p*-value		**0.031**	0.32	**0.001**
	% of control		**170%**	120%	**259%**
**2D**	Mean ± SEM	0.763 ± 0.071	0.801 ± 0.056	0.757 ± 0.041	0.878 ± 0.140
	*p*-value	0.264	0.68	0.96	0.43
	% of control		105%	99%	115%
**2C**	Mean ± SEM	0.825 ± 0.071	0.834 ± 0.057	0.780 ± 0.046	0.930 ± 0.137
	*p*-value		0.93	0.64	0.47
	% of control		101%	95%	113%
**3U**	Mean ± SEM	0.863 ± 0.070	0.904 ± 0.062	0.862 ± 0.053	0.981 ± 0.150
	*p*-value		0.66	0.99	0.43
	% of control		105%	100%	114%
**4G**	Mean ± SEM	0.981 ± 0.076	1.050 ± 0.091	0.955 ± 0.066	1.222 ± 0.218
	*p*-value		0.57	0.81	0.20
	% of control		107%	97%	125%
**5S**	Mean ± SEM	1.997 ± 0.110	2.158 ± 0.139	2.051 ± 0.120	2.249 ± 0.332
	*p*-value		0.37	0.76	0.20
	% of control		108%	103%	118%
**6Rot**	Mean ± SEM	1.486 ± 0.059	1.610 ± 0.087	1.531 ± 0.076	1.752 ± 0.202
	*p*-value		0.24	0.65	0.09
	% of control		108%	103%	104%
**7Gp;U**	Mean ± SEM	5.025 ± 0.433	4.672 ± 0.314	4.495 ± 0.386	4.990 ± 0.571
	*p*-value		0.52	0.38	0.96
	% of control		93%	89%	99%
**8AMA**	Mean ± SEM	0.039 ± 0.009	0.018 ± 0.007	0.028 ± 0.010	0.001 ± 0.001
	*p*-value		0.098	0.47	**0.037**
	% of control		47%	72%	1%
**CIV**	Mean ± SEM	4.499 ± 0.563	4.479 ± 0.382	4.278 ± 0.391	4.840 ± 0.854
	*p*-value		0.98	0.75	0.75
	% of control		100%	95%	108%
**1-L/P**	Mean ± SEM	0.817 ± 0.016	0.733 ± 0.028	0.791 ± 0.018	0.629 ± 0.039
	*p*-value		**0.014**	0.30	**0.006**
	% of control		**90%**	97%	**77%**

**Table 3:** Platelet mitochondrial respiration and fatty acid oxidation in EA patients by protocol RP2 (O_2_ flux/CS (pmol/s/IU)). Legend: see Figure 3.

Parameter	Group	Control	EA-All pts	EA-0 pts	EA-1 pts
	N	18	14	9	5
**ce**	Mean ± SEM	0.529 ± 0.028	0.610 ± 0.032	0.624 ± 0.044	0.586 ± 0.045
	*p*-value		0.063	0.069	0.34
	% of control		115%	118%	111%
**Dig**	Mean ± SEM	0.024 ± 0.010	0.031 ± 0.010	0.030 ± 0.012	0.033 ± 0.017
	*p*-value		0.61	0.70	0.67
	% of control		120%	125%	137%
**1OctM0.1**	Mean ± SEM	0.142 ± 0.017	0.211 ± 0.026	0.173 ± 0.014	0.280 ± 0.061
	*p*-value		**0.030**	0.26	**0.006**
	% of control		**149%**	122%	**197%**
**2D**	Mean ± SEM	0.247 ± 0.024	0.269 ± 0.024	0.252 ± 0.016	0.299 ± 0.065
	*p*-value		0.54	0.90	0.37
	% of control		109%	102%	121%
**2D;c**	Mean ± SEM	0.307 ± 0.032	0.294 ± 0.032	0.265 ± 0.021	0.346 ± 0.081
	*p*-value		0.78	0.38	0.60
	% of control		96%	86%	113%
**3M2**	Mean ± SEM	0.599 ± 0.055	0.573 ± 0.054	0.510 ± 0.021	0.687 ± 0.142
	*p*-value		0.74	0.24	0.48
	% of control		96%	85%	115%
**4P**	Mean ± SEM	0.688 ± 0.062	0.707 ± 0.065	0.628 ± 0.027	0.849 ± 0.169
	*p*-value		0.83	0.49	0.28
	% of control		103%	91%	123%
**5G**	Mean ± SEM	0.757 ± 0.073	0.811 ± 0.090	0.694 ± 0.042	1.021 ± 0.223
	*p*-value		0.64	0.55	0.15
	% of control		107%	92%	135%
**6S**	Mean ± SEM	1.689 ± 0.088	1.920 ± 0.120	1.816 ± 0.098	2.107 ± 0.289
	*p*-value		0.12	0.37	0.072
	% of control		114%	108%	125%
**7U**	Mean ± SEM	1.747 ± 0.101	1.992 ± 0.142	1.860 ± 0.113	2.229 ± 0.339
	*p*-value		0.16	0.50	0.074
	% of control		114%	107%	128%
**1-L/Pc**	Mean ± SEM	0.572 ± 0.056	0.322 ± 0.045	0.379 ± 0.054	0.207 ± 0.041
	*p*-value		**0.003**	**0.038**	**0.005**
	% of control		**56.3%**	**66.3%**	**36%**

**Table 4:** Antioxidants and TBARS in patients with Ebstein anomaly.

Parameters	Groups	Control	EA-All pts	EA-0 pts	EA-1 pts
	N	13	11	9	5
**Platelets (pmol/10^9^ cells)**					
**γ** **-Tocopherol**	Mean ± SEM	153.6 ± 29.8	53.2 ± 11.4	45.3 ± 12.0	67.3 ± 24.3
	*p*-value		**0.003**	**0.009**	0.11
	% of control		**35%**	**29%**	**44%**
**α** **-Tocopherol**	Mean ± SEM	4624 ± 708	7772 ± 856	8472 ± 1264	6513 ± 546
	*p*-value		**0.009**	**0.010**	0.14
	% of control		**168%**	**183%**	**141%**
**CoQ_10-Total_**	Mean ± SEM	84.6 ± 5.9	58.2 ± 7.5	62.7 ± 10.1	49.9 ± 10.9
	*p*-value		**0.009**	**0.056**	**0.011**
	% of control		**69%**	**74%**	**59%**
**Plasma (µmol/L)**					
**γ** **-Tocopherol**	Mean ± SEM	1.55 ± 0.19	1.341 ± 0.192	1.242 ± 0.231	1.520 ± 0.360
	*p*-value		0.45	0.32	0.94
	% of control		87%	80%	98%
**α** **-Tocopherol**	Mean ± SEM	34.70 ± 1.7	33.83 ± 1.91	36.30 ± 2.26	29.38 ± 2.68
	*p*-value		0.72	0.58	0.13
	% of control		97%	105%	85%
**β** **-Carotene**	Mean ± SEM	0.357 ± 0.042	0.400 ± 0.042	0.431 ± 0.047	0.344 ± 0.083
	*p*-value		0.48	0.27	0.88
	% of control		112%	121%	96%
**CoQ_10-Total_**	Mean ± SEM	0.485 ± 0.030	0.409 ± 0.047	0.411 ± 0.059	0.403 ± 0.086
	*p*-value		0.17	0.23	0.27
	% of control		84%	85%	83%
**TBARS**	Mean ± SEM	4.74 ± 0.18	4.90 ± 0.17	4.83 ± 0.10	5.03 ± 0.46
	*p*-value		0.52	0.73	0.49
	% of control		103%	102%	106%
**α** **-Toc/** **γ** **-Toc**	Mean ± SEM	25.70 ± 2.30	32.00 ± 4.70	37.40 ± 6.50	22.20 ± 3.8
	*p*-value		0.22	**0.054**	0.48
	% of control		126%	**147%**	87%

## Data Availability

The original contributions presented in this study are included in the article. Further inquiries can be directed to the corresponding author.
